# Population pharmacokinetics of haloperidol in terminally ill adult patients

**DOI:** 10.1007/s00228-017-2283-6

**Published:** 2017-07-05

**Authors:** L. G. Franken, R. A. A. Mathot, A. D. Masman, F. P. M. Baar, D. Tibboel, T. van Gelder, B. C. P. Koch, B. C. M. de Winter

**Affiliations:** 1 Department of Hospital Pharmacy, Erasmus Medical Centre, Wytemaweg 80, 3015 CN Rotterdam, The Netherlands; 2 Hospital Pharmacy – Clinical Pharmacology, Academic Medical Centre, Amsterdam, The Netherlands; 3 Palliative Care Centre, Laurens Cadenza, Rotterdam, The Netherlands; 4 Intensive Care, Department of Paediatric Surgery, Erasmus MC-Sophia Children’s Hospital, Rotterdam, The Netherlands; 5 Pain Expertise Centre, Erasmus MC-Sophia Children’s Hospital, Rotterdam, The Netherlands

**Keywords:** Haloperidol, Pharmacokinetics, Delirium, Palliative care

## Abstract

**Purpose:**

Over 80% of the terminally ill patients experience delirium in their final days. In the treatment of delirium, haloperidol is the drug of choice. Very little is known about the pharmacokinetics of haloperidol in this patient population. We therefore designed a population pharmacokinetic study to gain more insight into the pharmacokinetics of haloperidol in terminally ill patients and to find clinically relevant covariates that may be used in developing an individualised dosing regimen.

**Methods:**

Using non-linear mixed effects modelling (NONMEM 7.2), a population pharmacokinetic analysis was conducted with 87 samples from 28 terminally ill patients who received haloperidol either orally or subcutaneously. The covariates analysed were patient and disease characteristics as well as co-medication.

**Results:**

The data were accurately described by a one-compartment model. The population mean estimates for oral bioavailability, clearance and volume of distribution for an average patient were 0.86 (IIV 55%), 29.3 L/h (IIV 43%) and 1260 L (IIV 70%), respectively. This resulted in an average terminal half-life of haloperidol of around 30 h.

**Conclusion:**

Our study showed that the pharmacokinetics of haloperidol could be adequately described by a one-compartment model. The pharmacokinetics in terminally ill patients was comparable to other patients. We were not able to explain the wide variability using covariates.

## Introduction

In palliative care, (terminal) delirium is a frequently seen symptom with over 80% of the advanced cancer patients experiencing delirium in their final days [1]. Although randomised clinical trials are scarce, most guidelines and specialist consider haloperidol, a typical/classic antipsychotic, to be the first-line treatment of delirium [2–4]. Haloperidol is metabolised by several different pathways, involving cytochrome P450 (CYP), carbonyl-reductase and uridine diphosphoglucose glucuronosyltransferases (UGT) enzymes. Glucuronidation appears to be the major metabolic route followed by the reversible reduction of haloperidol to reduced haloperidol and CYP-mediated oxidation by CYP3A and CYP2D6.

The dose of haloperidol is determined based on the clinical effect. As haloperidol can cause motor (or extrapyramidal) and cardiovascular adverse/side effects, it is normally started at a low dose (0.5–2.0 mg) and increased slowly until the desired effect is reached. This can be disadvantageous in the case of refractory symptoms when rapid symptom relief is required. In addition, haloperidol has a relative long terminal half-life (*t*_1/2_) of approximately 20 h causing steady state to be reached after 4 to 5 days. Patients could therefore potentially benefit if an individualised dose is determined beforehand. In palliative patients, this is even more important, as rapid symptom relief is essential in the last phase of life.

Several studies on the pharmacokinetics of haloperidol in healthy volunteers or psychiatric patients showed large interindividual variability (IIV) [5–11]. As haloperidol has a moderate hepatic extraction ratio of 0.3–0.7, its metabolism may be influenced by hepatic blood flow as well as intrinsic enzyme activity and protein binding [7, 12, 13]. Therefore, the IIV in palliative care patients may be even more pronounced as these patients may suffer from decreased blood flow, altered plasma protein levels and possibly hepatic dysfunction [14]. We performed a population pharmacokinetic study in terminally ill patients to gain more insight into the pharmacokinetics in this population and to find clinically relevant parameters for dose individualisation.

## Methods

The study was performed in accordance with the principles of the Declaration of Helsinki and its later amendments. Ethical approval was obtained from the Medical Ethics Committee of the Erasmus University Medical Centre, Rotterdam, and all patients provided written informed consent.

## Data

Data was collected in the palliative care centre, Laurens Cadenza Zuid in Rotterdam, The Netherlands, during 2 years. Patients were eligible if they had a terminal illness, survival prognosis of more than 2 days and less than 3 months and administration of haloperidol. Informed consent was asked shortly after admittance to the palliative care centre, and included patient were followed until the time of death, unless informed consent was withdrawn at any point. Only patients who can give their consent themselves were asked for consent. The patients gave consent after contacting their family about the study. The partner/legal representative cosigned the informed consent form as witness of the consent. The investigator kept close contact with the patients and their family during the study. In the terminal phase, when the patient cannot be asked anymore, all aspects of the study were communicated with the family.

Haloperidol was given to treat deliria and was dosed in accordance with the current guidelines [4]. Haloperidol was administered orally (either as tablets or as a liquid formulation) or via subcutaneous bolus injection. The exact times of administration were recorded in the patient record. Any concomitant medication was also registered in the patient’s record. Demographic characteristics (age, gender, weight, race, primary diagnosis and time of death) were extracted from the electronic medical records.

## Blood sampling and assay

Blood samples were collected randomly via sparse sampling in both the pre-terminal and terminal phases on average at one to two occasions during the day, with a maximum of ten a week, 0.5 to 1 mL of blood. The moment of sampling is not strictly defined, but follows the clinical condition of the patient. For example, before and after the change to another administration route or in case of inadequate effect of a drug, blood will be sampled. Blood for clinical chemistry is routinely sampled by venous puncture. For this study, sampling is as much as possible combined with those venous punctures. Otherwise, sampling from an indwelling venous catheters is preferred. With the terminal phase being the last hours or days before death, a patient becomes bedbound, semi-comatose, and is not able to take more than sips of fluid [15]. During the terminal phase, blood is sampled only from an indwelling venous catheter. This method prevents repeated puncturing and causes minimal or no discomfort to the patient. The samples were centrifuged after which the plasma was collected and stored at −80 °C until analysis. The blood samples were preferably collected at the same time as sampling for clinical chemistry (standard of care). In the clinical chemistry samples, serum levels of albumin, creatinine, urea, bilirubin, gamma-glutamyl transpeptidase (GGT), alkaline phosphatase (ALP), alanine transaminase (ALT), aspartate transaminase (AST) and C-reactive protein (CRP) were determined.

Haloperidol concentrations were analysed in the plasma samples using LC-MS/MS with electrospray ionisation in the positive ionisation mode on a Shimadzu LC-30 (Nishinokyo-Kuwabaracho, Japan) system coupled to an AB Sciex (Framingham, MA, USA) API5500Q MS. Seventy-five-microlitre acetonitrile/methanol 84:16 (*v*/*v*%) containing the internal standard haloperidol-d4 was added to 10 μL of patient’s plasma to precipitate proteins. Afterwards, samples were vortexed and stored at −20 °C for 30 min to optimise protein precipitation, vortexed again and centrifuged. Three-microlitre sample was injected onto a Thermo Scientific Hypersil Gold (50 × 2.1 mm, 1.9 μm) column. A stepwise chromatographic gradient was applied using 0.05% ammonium formate/0.10% formic acid in water as mobile phase A and acetonitrile as mobile phase B. The flow rate was 0.4 mL/min and the column was kept at 40 °C. Using multiple reaction monitoring (MRM) with positive ionisation mode, haloperidol was measured as [M + H]^+^ using the mass transition 376.1/165.1. The lower limit of quantification was 0.5 μg/L and the method was validated over a range of 0.5–125 μg/L. The accuracies ranged from 93.5 to 107.4%.

## Software

Pharmacokinetic analysis was conducted by the NONMEM® Version 7.2 (ICON Development Solutions, Ellicott City, MD), PsN® (version 4.4.8), R (version 3.3.0) and Pirana (version 2.9.2).

## Population pharmacokinetic method

Log-transformed plasma haloperidol concentrations were used and the bioavailability of subcutaneous haloperidol was assumed to be 100% [16]. One-compartment and two-compartment models were tested for haloperidol using the first-order conditional estimation method with interaction (FOCE + I). To account for the two different administration routes (oral and subcutaneously), the ADVAN5 subroutine was used. Interindividual variability (IIV) was assessed on each parameter using an exponential model. Residual variability was included as a combined error model.

As weight has been shown to be a covariate in other haloperidol pharmacokinetic models, and as the relationship between body weight and clearance is well documented, this effect was tested using algometric scaling [11, 17]. In the covariate analysis, demographic and disease characteristics including weight, age, gender, primary diagnosis, renal function (plasma creatinine and plasma urea), hepatic function (plasma levels of bilirubin, GGT, ALP, ALT and AST), C-reactive protein (CRP), albumin and the concomitant use of CYP2D6 and CYP3A inductors and inhibitors were evaluated for their influence on clearance (CL), volume of distribution (*V*d) and bioavailability (*F*). Time to death (TTD) was also evaluated as a covariate. This parameter cannot be used as a covariate parameter for a priori prediction of individual pharmacokinetic changes but it may give insight into quantitative changes at the end of life that are not predicted by standard blood chemistry tests. The relationship between covariates and individual estimates was first investigated graphically and was further tested with a forward inclusion, backward elimination approach with *P* values of 0.05 and 0.001, respectively.

Continuous covariates were normalised to the population median values and incorporated as power model functions (Eq. 1). Categorical covariates were transformed to binary covariates and incorporated as shown in Eq. 2.

1 $$ {\theta}_i={\theta}_{\mathrm{pop}}\kern0.5em \times \kern0.62em {\left(\frac{{\mathrm{cov}}_i}{{\mathrm{cov}}_m}\right)}^{\theta \operatorname{cov}} $$

2 $$ {\theta}_i={\theta}_{\mathrm{pop}}\kern0.5em \times \kern0.5em {\theta_{\mathrm{cov}}}^{{\mathrm{cov}}_i}, $$

with *θ*_*i*_ being the individual model predicted pharmacokinetic parameter (e.g. clearance) for an individual with covariate value cov_*i*_, *θ*_pop_ being the population estimate for that parameter, cov_*m*_ representing the median covariate value and *θ*_cov_ the covariate effect. In the equation, for categorical covariates, cov_*i*_ is either 1 or 0.

To evaluate the time to death (TTD) as a covariate, time-dependency of the parameters was modelled as a first-order process given to following equation (Eq. 3).

3 $$ {\theta}_i={\theta}_{\mathrm{pop}}-{\theta}_{\Delta}\kern0.5em \times \kern0.5em  \exp \left(-{\theta}_{\mathrm{rate}}\kern0.5em \times \kern0.5em \mathrm{TTD}\right), $$

in which *θ*_Δ_ is the change in parameter value from its initial value and *θ*_rate_ is a first*-*order rate constant determining the rate with which the parameter value changes over time.

## Model evaluation

Intermediate models were evaluated based on minimum OFV parameter precision, error estimates, shrinkage values and visual inspection of the goodness of fit plots. A bootstrap with 500 runs was performed on the final model to evaluate the validity of the parameters estimates and their corresponding 95% percentile ranges. The final model was evaluated with a normalised prediction distribution error (NPDE) analysis. NPDE is a simulation-based diagnostics which can be used to evaluate models developed on datasets with variable dosing regimens. The analytical value of this method has been previously described by Comets et al. [18].

## Simulations

To give an illustration of the effect of dose on the plasma concentrations of haloperidol and the variability, deterministic simulations were performed. The haloperidol plasma concentrations were simulated over a time course of 72 h in which six subcutaneous doses were administered every 12 h. To show the interpatient variability, the mean and 90% confidence interval are shown graphically.

## Results

A total of 28 terminally ill patients were included in the study. Their median age was 69.5 years (range 43–93), 54% were male and all patients had advanced malignancy as primary diagnosis with the majority (87%) having epithelial tissue as the primary malignant site. On average at one to two occasions during the day, with a maximum of ten a week, 0.5 to 1 mL of blood was collected from the patient by vena puncture or indwelling venous catheter to determine drug concentrations.

An overview of all patient characteristics is given in Table 1. Oral doses of haloperidol ranged from 0.5 to 2 mg a day and subcutaneous doses ranged from 0.5 to 5 mg a day. A total of 86 blood samples were collected. 26.7% of the concentrations were below the quantification limit (BQL). On closer inspection, half of these plasma concentrations were measured in samples taken over 200 h after the last haloperidol dose. Discarding these resulted in 14.6% BQL data left within 200 h after the last dose. As this is still more than 10%, the M3 method of handling BQL data was used to estimate if BQL data were indeed below the lower limit of quantification of 0.5 mg/L [19, 20]. As this resulted in similar parameter estimates but stability issues, the M1 method (of discarding the BQL data) was used for the final model.

**Table 1:** Patient characteristics over the time course of the study

Characteristics	*N* = 28
Age, years (median, range)	69.5 (43–93)
Male, *n* (%)	15 (53.6)
Female, *n* (%)	23 (51.1)
Weight, kg (median, range)	67 (35–108)
Ethnic origin, *n* (%)	
Caucasian	26 (92.9)
Afro-Caribbean	2 (7.1)
Primary diagnosis, *n* (%)	
Neoplasm	28 (100)
Epithelial tissue	25 (89.3)
Connective tissue	1 (3.4)
Haematological	1 (3.4)
Not specified	1 (3.4)
Blood chemistry, serum levels at admission (median, range)	
Albumin, g/L	25 (13–39)
Creatinine, μmol/L	79 (20–673)
Urea, mmol/L	8.3 (1.5–43.4)
Bilirubin, μmol/L	9 (3–256)
Gamma-glutamyl transpeptidase, U/L	57 (7–1055)
Alkaline phosphatase, U/L	117 (20–2117)
Alanine transaminase, U/L	14 (7–116)
Aspartate transaminase, U/L	32 (13–396)
C-reactive protein, U/L	115 (1–346)
Patients using dexamethasone^a^, *n* (%)	11 (39.3)
Patients using citalopram^a^, *n* (%)	1 (3.4)
Patients using paroxetin^a^, *n* (%)	5 (17.9)
Duration of stay^b^, days (median, range)	18.6 (1.5–176.6)
Blood samples collected^c^, *n* (median, range)	3 (1–9)

^a^During any moment while receiving haloperidol treatment

^b^From start of first haloperidol dose until time of death

^c^From the start of the first haloperidol dose

## Structural model

The data were best described by a one-compartment model with an additive residual error on logarithmic transformed concentrations. Since there was limited data available in the absorption phase, the absorption constants (*K*a) could not be estimated. We therefore derived this value from literature, and as there was no literature available for the absorption time of subcutaneous injection of the iv formulation, intramuscular administration was used as a reference [21]. Changing this assumption to half of the absorption rate did not affect the other parameters, which indicates that the model is stable and not influenced by this assumption. IIV was included on the parameters CL, *F* and *V*d. As the IIV on CL and *F* showed a high degree of correlations (99%), these were fixed to unity with the addition of an extra theta.

## Covariate analysis

Allometric scaling was tested both with an estimated scaling factor for CL and *V*d as well as fixed scaling factors of 0.75 and 1, respectively (Eq. (4)). As the values of 0.75 and 1 lay within the 95 confidence intervals of the estimated scaling factors, and because estimating the scaling factors did not significantly improve the model fit, fixed values of 0.75 and 1 were used. Including allometric scaling significantly improved the model fit (ΔOFV 7.47, *P* < 0.05) and decreased the IIV on *V*d with 13%. If the weight of an individual was unknown, the median weight of the population (67 kg) was imputed. This was the case for 35% of the study population.

4 $$ {\theta}_i={\theta}_{\mathrm{pop}}\times {\left(\frac{{\mathrm{WGT}}_i}{{\mathrm{WGT}}_{\mathrm{median}}}\right)}^{\mathrm{scaling}\ \mathrm{factor}} $$

Besides bodyweight, plasma bilirubin concentration was significant on the volume of distribution in the forward inclusion. This resulted in statistically significant improvement of the model fit, with a drop in objective function value (OFV) of 7.22 points and a decrease in IIV on *V*d from 61 to 43.2%, thereby explaining 31% of the IIV on *V*d. Both parameters were not significant in the backward elimination. After inspecting the individual influence on the decrease in OFV using sharkplots, it was shown that for both covariates, there were two very influencing individuals, with just one individual being responsible for reaching the statistical significance. Bodyweight and plasma bilirubin were therefore not included in the final model. An overview of all parameter estimates is given in Table 2.

**Table 2:** Parameter estimates of the final model and bootstrap analysis

Parameter	Final model	RSE %	Shrinkage %	Bootstrap of the final model		
				Average	95% percentile (lower)	95% percentile (upper)
Structural parameters						
*F*	0.861	18	–	0.76	0.50	0.97
*K*a _oral route_	0.236^a^	–	–	–	–	–
*K*a _subcutaneous route_	20^a^	–	–	–	–	–
CL (L/h)	29.3	11	–	27.6	19.8	32.4
*V*d (L)	1260	19	–	1283	794	1982
IIV (%)						
*F*	55	43	37	56	20	218
CL	43	34	29	48	22	131
*V*d	70	21	31	65	32	108
Residual variability						
	0.258	22	19	0.238	0.144	0.353

*F* bioavailability, *K*a absorption rate, CL clearance, *V*d volume of distribution

^a^The *K*a values were fixed to literature values as there were no pharmacokinetic studies on haloperidol solution via subcutaneous administration; this *K*a was calculated from a *T*max of 20 min which is the literature value of the intramusculair route [11]

## Model evaluation

**Fig. 1:**
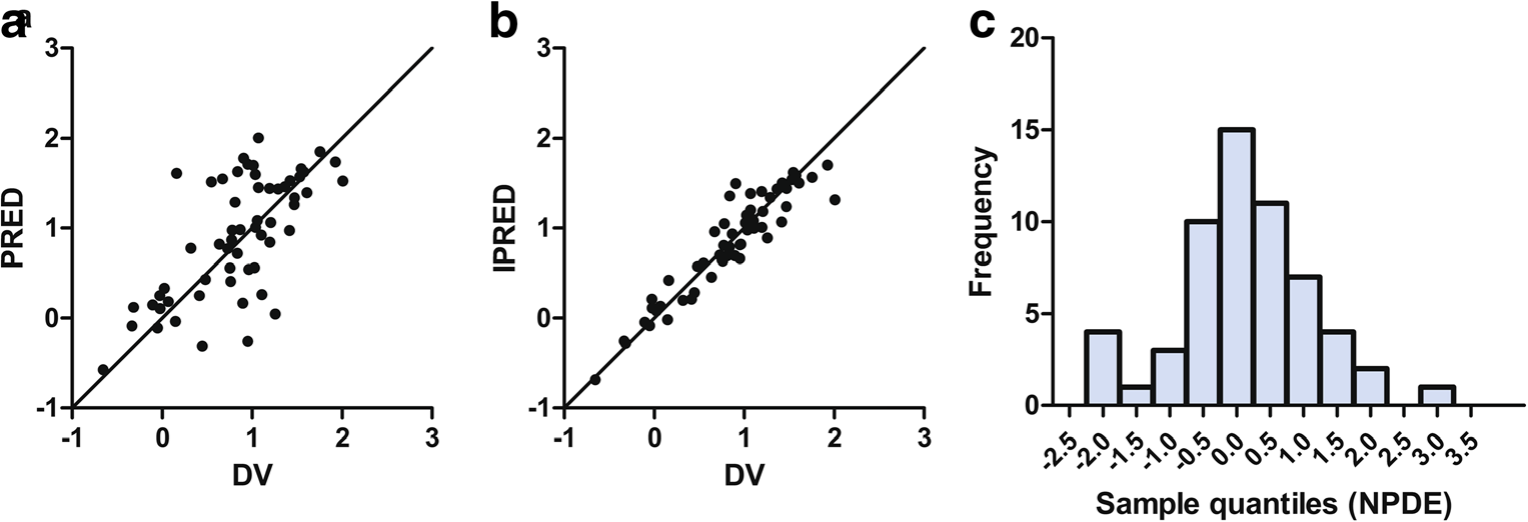
Goodness of fit plots of the final model. Population predictions (PRED) versus observations of haloperidol (**a**), individual predictions (IPRED) versus observations of haloperidol (**b**) and the normalised prediction distribution error (NPDE) distribution plot (**c**) for of the final model showing NPDE quantiles

Figure 1a, b shows that both the population predictions and individual predictions were evenly distributed around the line of unity when plotted against the observations. A bootstrap analysis of the final model was performed to obtain 95% percentile ranges for all parameters. Results of the bootstrap are shown in Table 2. Evaluation of the predictive performance by NPDE analysis showed accurate predictive ability, with the distribution of the NPDEs not significantly deviating from a normal distribution (with a global adjusted *P* values of 0.4), and the majority of the NPDEs laying between the values −2 and 2 (Fig. 1c).

## Simulations

**Fig. 2:**
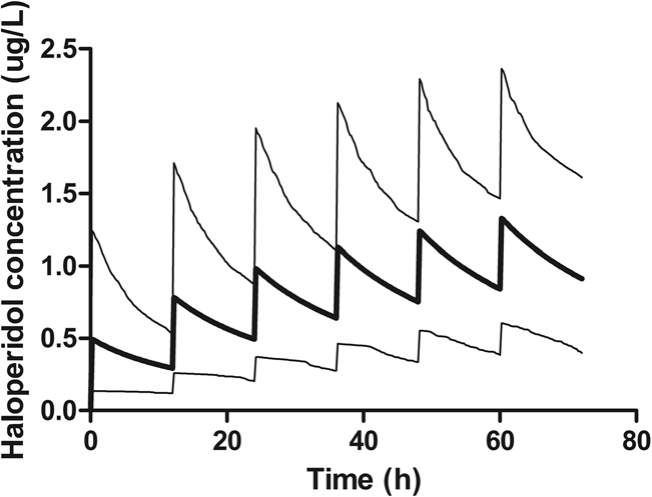
Simulated plasma profiles of haloperidol 0.5 mg every 12 h. The mean concentration and 90% confidence interval are presented from a simulation of 1000 patients

The effect of 0.5 mg of subcutaneously administered haloperidol every 12 h is shown in Fig. 2. The plasma concentration is very variable between patients.

## Discussion

To our knowledge, this is the first population pharmacokinetic study of haloperidol in terminally ill adult patients. We were able to describe the pharmacokinetics of haloperidol with adequate accuracy using a sparse sampling method. The simulations show the high interpatient variability in the pharmacokinetics and the effect of the long terminal half-life of haloperidol. The *t*_1/2_ of around 30 h from our study means it would take a long time to reach steady-state levels and it would take about 6 days to completely eliminate a single haloperidol dose from the body.

The covariate analysis did not result in any significant covariates. Initially, body weight and plasma bilirubin levels seemed to be correlated; however, as this was mainly due to influence of one or two individuals, these were not included as a covariate in the final model. Body weight was shown to be correlated in other studies [11, 17]. In our study, body weight was not registered for all patients and can vary a lot in the terminal phase, which might explain the lack of this correlation in this group of patients. It does not seem likely that hepatic dysfunction is correlated with the volume of distribution. A correlation with clearance seems more logical as haloperidol has a moderate hepatic extraction ratio of 0.3–0.7. The fact that none of the hepatic markers showed a correlation with clearance may be because of the limited data in our study or because of the fact that the liver has a high over capacity for metabolising drugs. Furthermore, the fact that none of the co-medication showed a correlation with clearance may also be due to the low number of patients using concomitant medication at the time of haloperidol use. This is common in the palliative population as the majority of medication is discontinued in the palliative phase. In addition, a lack of effect of co-medication may have been expected as there are several different metabolic pathways involved in the metabolism of haloperidol; therefore, co-medication that affects only one of these routes will most likely have no effect on the overall clearance of haloperidol.

There have been previous studies in patients with schizophrenia which showed estimates for clearance which were 1.5 to more than 2 times higher than the 29.3 L/h found in our study, when corrected for bioavailability of 86% [10, 11]. However, as both studies also reported higher estimates for the total volume of distribution, these together result in *t*_1/2_ values of 25 and 39.5 h which is comparable to the *t*_1/2_ of 30 h found in our study. It seems reasonable that terminally ill patients have a lower clearance and a lower volume of distribution compared to schizophrenia patients, who on average are younger and are less physically ill. Another difference with the study by Pilla Redy et al. is that they found haloperidol to be best described by a two-compartment model. This can possibly be explained by the fact that in our study, we had more sparse data and were therefore unable to accurately describe a peripheral compartment and inter-compartmental clearance. This is supported by the fact that their study had over 500 samples which still resulted in a broad 95% CI for the peripheral volume of distribution.

Both studies had weight incorporated in their final model. Unfortunately, one of the limitations of this study is that for about one third of the patients, the weight was unknown, and in fact, if the weight was known, this was a single value reflecting the weight at admission rather than several measurements over the study period. One of the reasons for the lack of data on weight is that almost none of the drugs given in the hospice setting were based on the patient’s weight, and therefore, it was unnecessary for clinical practice to collect data on weight. Another reason is that doctors and nurses were reluctant to weigh patients as it could be disturbing for the patient to be faced with their weight loss. There are several ways to handle missing covariate data in population pharmacokinetic analysis [22]. We tried to incorporate these methods in our model. However, as we did not find a correlation between weight and any of the other known covariates, a method to handle missing data was not feasible. We also tested a model with different population values or IIV values for known and unknown weights. This did not result in significant improvements and resulted in large shrinkage values and model instability due to the already sparse sample numbers, and it was therefore not feasible to use in the final model.

Probably, the most important limitation, in general, is the fact that an effective plasma concentration of haloperidol is still unknown. The study of Pilla Redy et al. showed that the overall EC50 value was 2.7 mg/L on an overall scale of schizophrenia, with considerably lower effective concentrations for the positive symptoms (0.5 mg/L) than the negative symptoms (31 mg/L). When we look at deliria, this may show more similarities with the positive symptoms of schizophrenia than the negative. However, the underlying cause in the case of (terminal) deliria is completely different, making it difficult to give any target concentrations for haloperidol in terminally ill population. Reference values of haloperidol are only based on schizophrenia dosing and more studies on delirium and PK/PD of haloperidol are needed.

Overall, this study showed that it is possible to describe the pharmacokinetics of haloperidol with adequate accuracy in terminally ill patients. We were not able to explain the variability in the pharmacokinetics using covariates. Before any recommendations can be made, more research is necessary, especially to the pharmacodynamic effects of haloperidol in this population as well as the possible effect of liver failure. The current Dutch guidelines recommends a dose of 0.5–2 mg subcutaneously every half an hour until an adequate effect is reached. Looking at the simulated plasma profiles in our study and keeping in mind the lack of any known effective dose, this seems a very reasonable recommendation, as the absorption constant of haloperidol is fast. The effect can probably be adequately assessed after half an hour and titrating up. Too fast dosing may result in adverse events that would take a long time to wear off due to the long terminal half-life.

In conclusion, this study describes the pharmacokinetics of haloperidol with adequate accuracy in terminally ill patients. More information on pharmacodynamics are needed to optimise dosing regimens of haloperidol in this patient group
